# Folding Landscape of Mutant Huntingtin Exon1: Diffusible Multimers, Oligomers and Fibrils, and No Detectable Monomer

**DOI:** 10.1371/journal.pone.0155747

**Published:** 2016-06-06

**Authors:** Bankanidhi Sahoo, Irene Arduini, Kenneth W. Drombosky, Ravindra Kodali, Laurie H. Sanders, J. Timothy Greenamyre, Ronald Wetzel

**Affiliations:** 1 Department of Structural Biology, University of Pittsburgh School of Medicine, Pittsburgh, PA, 15260, United States of America; 2 Pittsburgh Institute for Neurodegenerative Diseases, University of Pittsburgh School of Medicine, Pittsburgh, PA, 15260, United States of America; 3 Department of Neurology, University of Pittsburgh School of Medicine, Pittsburgh, PA, 15260, United States of America; University of Florida, UNITED STATES

## Abstract

Expansion of the polyglutamine (polyQ) track of the Huntingtin (HTT) protein above 36 is associated with a sharply enhanced risk of Huntington’s disease (HD). Although there is general agreement that HTT toxicity resides primarily in N-terminal fragments such as the HTT exon1 protein, there is no consensus on the nature of the physical states of HTT exon1 that are induced by polyQ expansion, nor on which of these states might be responsible for toxicity. One hypothesis is that polyQ expansion induces an alternative, toxic conformation in the HTT exon1 monomer. Alternative hypotheses posit that the toxic species is one of several possible aggregated states. Defining the nature of the toxic species is particularly challenging because of facile interconversion between physical states as well as challenges to identifying these states, especially *in vivo*. Here we describe the use of fluorescence correlation spectroscopy (FCS) to characterize the detailed time and repeat length dependent self-association of HTT exon1-like fragments both with chemically synthesized peptides *in vitro* and with cell-produced proteins in extracts and in living cells. We find that, *in vitro*, mutant HTT exon1 peptides engage in polyQ repeat length dependent dimer and tetramer formation, followed by time dependent formation of diffusible spherical and fibrillar oligomers and finally by larger, sedimentable amyloid fibrils. For expanded polyQ HTT exon1 expressed in PC12 cells, monomers are absent, with tetramers being the smallest molecular form detected, followed in the incubation time course by small, diffusible aggregates at 6–9 hours and larger, sedimentable aggregates that begin to build up at 12 hrs. In these cell cultures, significant nuclear DNA damage appears by 6 hours, followed at later times by caspase 3 induction, mitochondrial dysfunction, and cell death. Our data thus defines limits on the sizes and concentrations of different physical states of HTT exon1 along the reaction profile in the context of emerging cellular distress. The data provide some new candidates for the toxic species and some new reservations about more well-established candidates. Compared to other known markers of HTT toxicity, nuclear DNA damage appears to be a relatively early pathological event.

## Introduction

Susceptibility to Huntington’s disease (HD) is tightly linked to the expansion of a polyglutamine (polyQ) repeat sequence in the N-terminal segment of the protein huntingtin (HTT), but there is no consensus on how this repeat expansion endows the HTT protein with a toxic gain of function [1–3]. Short N-terminal fragments of HTT containing the polyQ domain, and in particular the HTT exon1 fragment, may well play a key role in the molecular mechanism of disease [4]. The prevailing model for the effect of repeat expansion is that it alters the folding landscape of these fragments, enriching the molecular ensemble in a conformational or assembly state whose interactions with key cellular targets produce cell dysfunction and death. One approach to experimentally addressing the challenge of expanded polyQ toxicity is to divide it into a series of more manageable individual problems. Here, we focus on the question of how the expansion of the polyQ sequence changes the folding / misfolding landscape of HTT exon1 fragments, and whether certain physical states are suppressed or elevated in response to repeat expansion.

**Fig 1:**
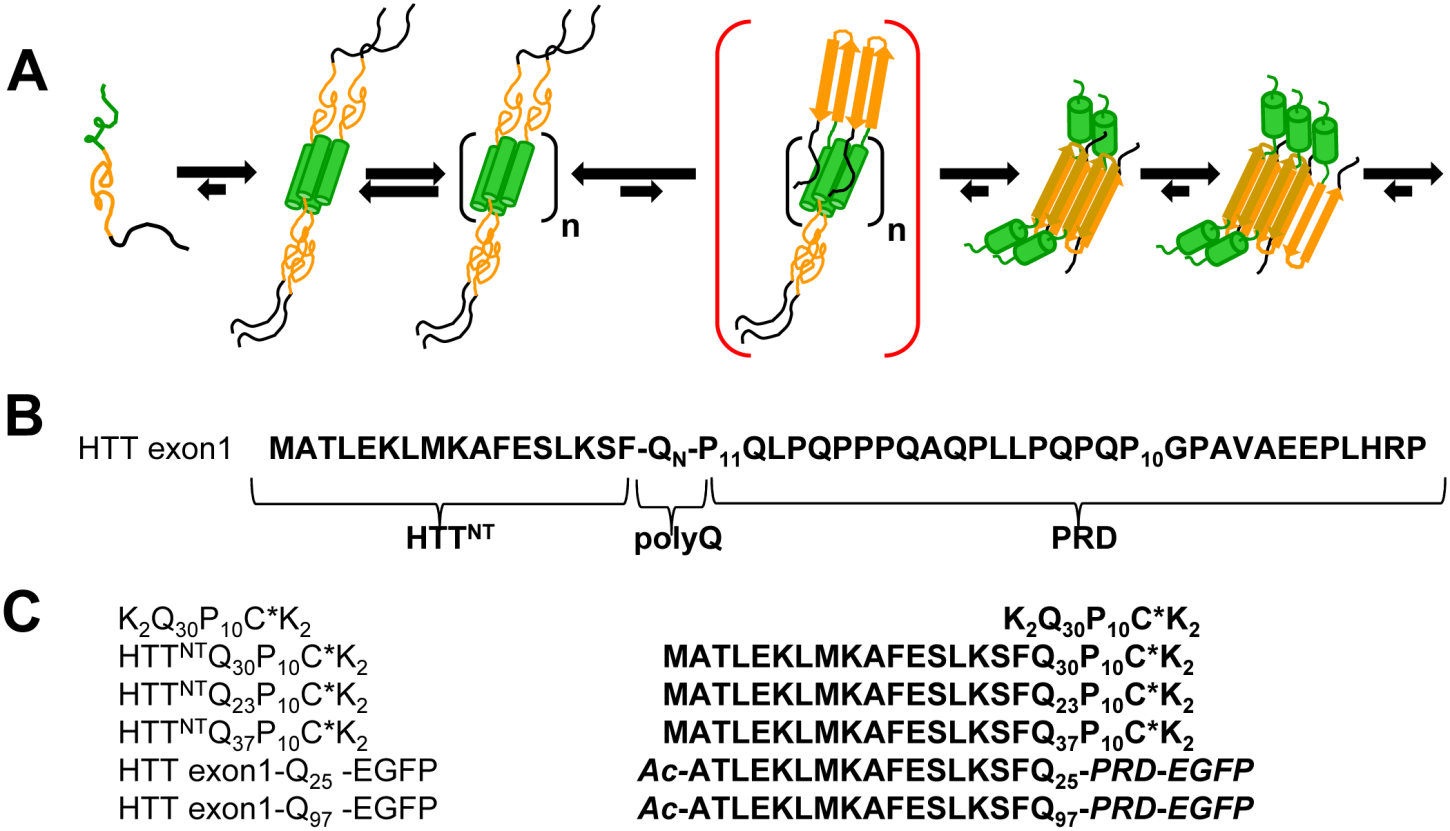
Structures and assembly mechanism of HTT exon1 polypeptides. A. Previously proposed mechanism of amyloid nucleation (HTT^NT^, green; polyQ, orange; PRD, black). B. Sequence of HTT exon1. C. Sequences of peptides studied. C* = Cys residue modified with Alexa Fluor 555 (see Materials and Methods).

Fragments like HTT exon1 consist of three segments, each of which is intrinsically disordered in the monomeric state [5] (Fig 1). Early experiments using a polyQ-specific antibody suggested a possible repeat length dependent conformational change within monomeric polyQ [6]. However, more recent work indicates a common compact coil state in both normal and pathological repeat lengths of polyQ [7], and a relatively short polyQ antibody epitope [8] whose binding strength to antibodies (and to possible cellular toxicity targets) depends on the number of independent epitopes within the chain [9,10]. Repeat length dependent formation of dimers or tetramers is another possible mechanism for repeat length dependence of disease, but there is no consensus on the multimeric state of HTT proteins. Purified, full-length HTT expressed in insect [11] or mammalian [12] cells has been shown by gels and/or by analytical ultracentrifugation (AUC) to contain appreciable amounts of dimer and higher multimers in addition to monomeric material. In contrast, full length HTT isolated from HD brain material, HD mouse model brains, or HD mouse model primary neurons has been shown to be monomeric [13]. Likewise, the low molecular weight form of purified, recombinantly expressed HTT exon1 has also been shown to be monomeric by AUC [14] and by size exclusion chromatography (SEC) coupled with multi-angle light scattering [10]. In contrast, however, AUC showed that fragments consisting of the N-terminal HTT^NT^ peptide (Fig 1) alone or followed by a Q_10_ sequence form distributions of monomer, tetramers, octamers and higher oligomers [15]. Recently, ion mobility mass spectrometry (MS) analysis revealed that in the gas phase the isolated HTT^NT^ fragment populates species ranging from monomer to tetramer [16]. Similar multimers of HTT exon1 might be the toxic species in a toxic gain-of-function mechanism, or might serve as vehicles by which mutant HTT fragments incapacitate WT fragments in a dominant negative disease mechanism [17]. Oligomers lacking β-structure have also been implicated as the phase within which polyQ amyloid fibril initiation takes place [5,15,18,19], one model for which is illustrated in Fig 1A.

Formation of amyloid-like aggregates is a common feature of physiological conditions involving Gln-rich proteins, from HD [20] to other expanded polyQ repeat diseases [21] to yeast prions [22]. Observations of large, polyQ-positive inclusions in neuronal nuclei in HD autopsy material [23] and animal models [24] implicated a role for protein aggregation in the HD disease process. This idea received additional support from reports of a repeat length dependence to aggregation *in vivo* [25] and *in vitro* [26,27] that recapitulates the repeat length dependence of disease risk and age-of-onset [3]. There are mixed results, however, on whether inclusions themselves might be the toxic species [28,29]. In any case, substantially smaller aggregated forms of HTT exon1 have been observed by electron microscopy (EM) and atomic force microscopy (AFM) when HTT exon1 is incubated *in vitro* [30], and have also been seen in fixed cells, cell extracts, and brain homogenates using native gels [31], SEC [32], glycerol gradient centrifugation [33], AFM [34], EM [34], and super-resolution fluorescence microscopy [35]. Most of these methods do not lend themselves to quantitation of aggregate size and concentration, however, and there have been only limited studies of the temporal linkage between the appearance of these smaller aggregates and the onset of pathology [31–34]. In one example, Leitman et al. identified a population of “soluble” oligomers associated with a degree of ER stress [33] that has been previously implicated in HD toxicity [36].

There are few tools available to study protein self-association at low physiological concentrations in living or fixed intact cells. Fluorescence microscopy has been used to visualize inclusions of fluorescently tagged proteins such as HTT exon1 in living cells [28]. These inclusions are multiple microns in diameter and may contain up to a billion protein molecules [37]. Smaller aggregates are not observable with this technique, but super-resolution fluorescence microscopy on fixed cells reveals clusters of amyloid fibrils in the range of a micron in length [38] that may contain up to 100,000 HTT molecules [37], and further refinements have led to visualization of smaller, single fibrils in the 1 μm length range [35]. Number and brightness analysis assessing *spatial* fluctuations of fluorescence intensity in confocal images revealed a mixture of monomers and small oligomers in the 5–15 range in early growth time points in mammalian cells expressing expanded polyQ HTT exon1 [39].

Fluorescence correlation spectroscopy (FCS) assessing *temporal* fluctuations in fluorescence intensity under conditions of free diffusion in solution [40] is a powerful and highly accurate technique for characterizing molecular sizes of fluorescently tagged molecules at relatively low concentrations in simple buffers and cell extracts [41] as well as in living cells [42]. However, FCS has been used only rarely in the study of protein aggregates and aggregation [43].

In the work described here we set out primarily to identify the molecular cast of characters populated by normal and polyQ repeat expanded HTT exon1 in aqueous systems. We use FCS to characterize and quantify the self-association states of HTT exon1 peptides both with chemically synthesized peptides *in vitro* and with expressed peptides in mammalian cell culture, and track how oligomerization states change with repeat length and incubation/growth time. We also characterize several markers of cell pathology over the same time frame. This work provides new insights into HTT exon1 self-association states and how their stabilities are influenced by polyQ repeat length. For expanded polyQ forms of HTT exon1, the work reveals the absence of detectible monomers but the presence of several small, self-associated forms, including dimers, tetramers and diffusible oligomers ranging from globular to fibrillary, that appear in the same time frame as the first identifiable cell pathology.

## Results

In FCS, a confocal microscope with laser optics that create a femptoliter focal volume is modified to allow time-dependent fluorescence fluctuations to be quantified with great sensitivity, accuracy and temporal resolution [40]. Using an autocorrelator, these fluctuations can be computationally modelled to derive the diffusion times and numbers of fluorescently labelled particles traversing the focal volume. These values can in turn be used to calculate molecular sizes and concentrations [40,41]. Although FCS is most accurate when characterizing systems at equilibrium [41], in principle it can be used to characterize aggregating systems, so long as aggregation times are slow in comparison to fluorescence data collection times under the conditions of measurement [40]. In spite of the complexity and relatively high viscosity of the intracellular environment, interpretable FCS data can even be obtained from measurements in living cells [42,44].

### HTT exon1 analogs in simple buffer: repeat-length and concentration dependent multimerization

**Fig 2:**
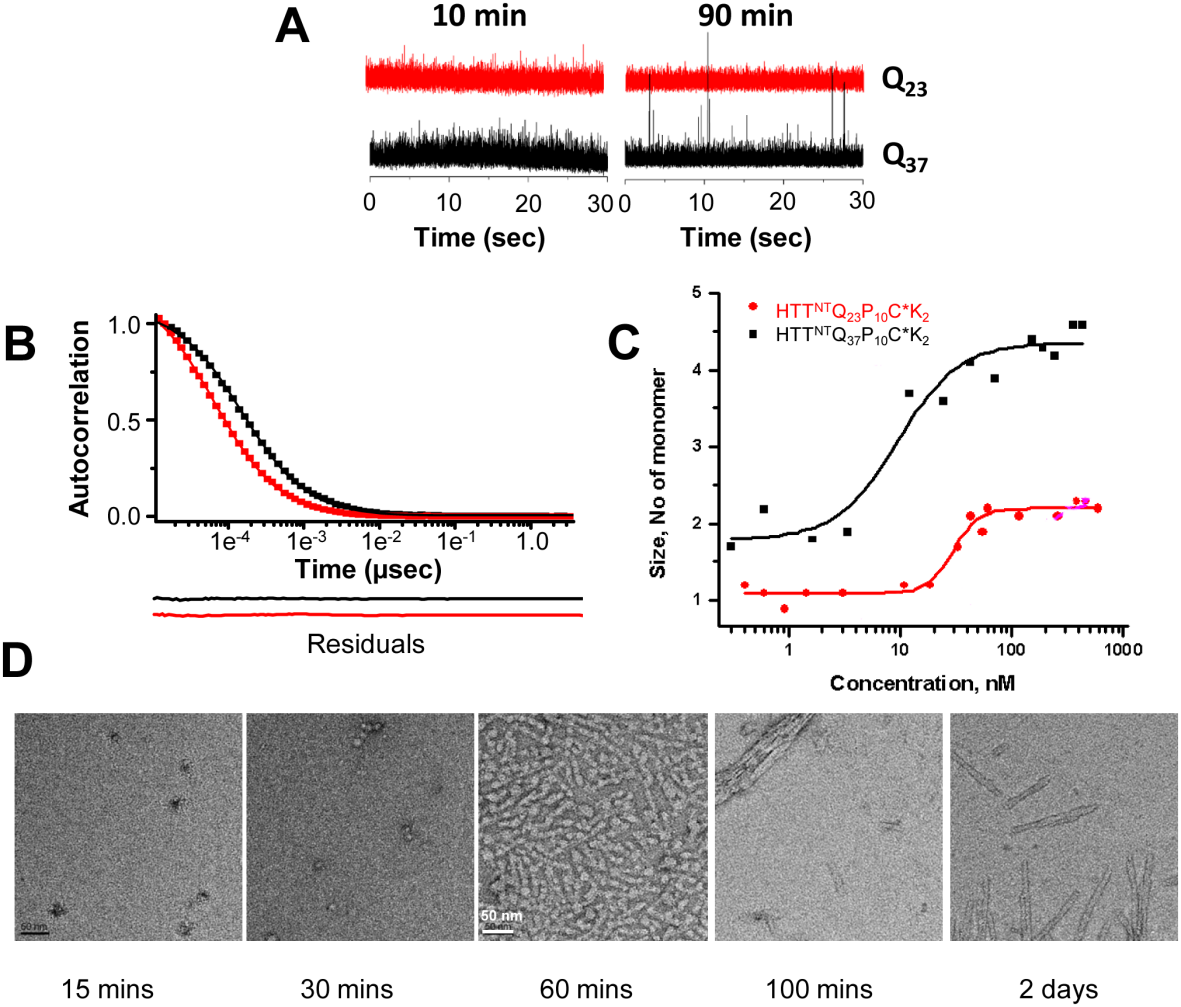
Self-assembly in PBS of chemically synthesized HTT exon1 analogs. A. Raw FCS time-dependent fluorescence fluctuations of HTT^NT^Q_23_P_10_C*K_2_ (red) and HTT^NT^Q_37_P_10_C*K_2_ (black). B. Autocorrelation functions for the 10 mins FCS data shown in A, with the data points as filled squares and solid lines representing the fits (see Materials and Methods) and the same color scheme as A. Lines below the graph show the residuals between the data points and the fit curve. C. Concentration dependence of molecular size estimated from diffusion times for HTT^NT^Q_37_P_10_C*K_2_ (■) and HTT^NT^Q_23_P_10_C*K_2_ (●). D. EM detail of different time points from the PBS incubation of a mixture of 2.0 μM HTT^NT^Q_37_P_10_K_2_. (More EM data is shown in S1 Fig.)

In order to map out the basic physical behavior of the HTT exon1 peptide system, we first investigated chemically synthesized HTT exon1 analogs containing the relatively small Alexa fluor-555 fluorescent dye covalently attached to a Cys residue placed near the C-termini of the peptides (Fig 1C). These analogs contained either a Q_23_ or a Q_37_ repeat, and a truncated proline-rich domain (PRD) consisting of a P_10_ sequence (Fig 1C). Importantly, such P_10_ analogs have been shown to behave similarly to full-length HTT exon1 in their aggregation properties, including details of the aggregation mechanism [18,37]. These peptides were disaggregated (Materials and Methods), suspended in PBS at 30 nM, and examined by FCS after a 10 min equilibration period at RT. The raw time-dependent fluorescence data (Fig 2A) show that both peptides exhibit fluorescence fluctuations within a fairly narrow intensity range, and the autocorrelation functions (Fig 2B) of these data are consistent with a single component. However, while the HTT^NT^Q_23_P_10_C*K_2_ molecule (Fig 2B, red curve) was found to exist as a monodisperse solution of monomers, the expanded polyQ analog HTT^NT^Q_37_P_10_C*K_2_ (Fig 2B, black curve) exists as a monodisperse solution of tetramers, with no monomers evident (Table 1).

**Table 1:** Summary of FCS data showing molecular sizes under various conditions.

Sample	time and/or conc.^a^	τ_D_1, μsec^b^	τ_D_2, μsec^b^	Size1^c^	Size2^c^
HTT^NT^Q_23_P_10_C*K_2_	30 nM, 10 mins	92	-	0.98	-
HTT^NT^Q_37_P_10_C*K_2_	30 nM, 10 mins	160	-	3.8	-
HTT^NT^Q_37_P_10_C*K_2_	30 nM, 90 mins	167	447	4.1	84
HTT^NT^Q_30_P_10_C*K_2_	150 nM, 10 mins	146		3.7	
K_2_Q_30_P_10_C*K_2_	150 nM, 10 mins	90		0.98	
K_2_Q_30_P_10_C*K_2_	150 nM, 40 mins	91		0.98	
K_2_Q_30_P_10_C*K_2_	150 nM, 90 mins	97		1.0	
GFP extr. (extract)	transient transfect.	150	-	1.0	-
HTT exon1-Q_25_-EGFP extr.	24 hrs	181	-	1.1	-
HTT exon1-Q_97_-EGFP extr.	0 hrs	270	-	3.4	-
HTT exon1-Q_97_-EGFP extr.	3 hrs	275	-	3.6	-
HTT exon1-Q_97_-EGFP extr.	6 hrs	281	503	3.9	21
HTT exon1-Q_97_-EGFP extr.	9 hrs	285	544	4.0	27
HTT exon1-Q_97_-EGFP extr.	12 hrs	295	1856	4.5	1096
HTT exon1-Q_97_-EGFP extr.	16 hrs	278	2138	3.7	1675
HTT exon1-Q_97_-EGFP extr.	24 hrs	266	2550	3.3	2843
GFP in cell	transient transfect.	1450	-	1.0	-
HTT exon1-Q_25_-EGFP in cell	6 hrs	1403	-	0.95	-
HTT exon1-Q_97_-EGFP in cell	6 hrs	2111	-	3.4	-

^a^Cells: post-induction growth times; peptides: concentrations and pre-measurement incubation time.

^b^Diffusion times of species #1 and (if any) species #2.

^c^Number of HTT exon1-like peptides per particle in species #1 and #2.

A particle’s diffusion time (τ_d_) in a particular buffer is influenced not only by mass but also by molecular shape. Although it seems highly unlikely that a conformational change within a Q_37_ HTT exon1-like monomer could account for a 400% increase in predicted mass, it was important to consider whether a repeat length dependent conformational change might be an alternative explanation for the substantially increased diffusion times observed for HTT^NT^Q_37_P_10_C*K_2_. After analysis, however, all the considerations below support multimerization and not shape changes being responsible for these increases. First, monomer-tetramer equilibria have already been described for HTT^NT^-containing peptides using analytical ultracentrifugation [15] and ion mobility MS [16]. Second, analysis shows that the diffusion times of both the Q_23_ and the Q_37_ peptides increase sigmoidally as peptide concentration increases (Fig 2C). This behavior is not consistent with a conformational change (which should be concentration independent), but is completely consistent with a simple mass action concentration effect on a monomer-dimer-tetramer self-association equilibrium. Third, estimates of the average molecular size in these samples by considering the statistical distribution of fluorescence intensities (“brightness analysis”; [40,45]) shows sizes consistent with FCS results. Thus, we used two different methods to analyze data from a HTT^NT^Q_23_P_10_C*K_2_ solution in the concentration range where the peptide was found by FCS diffusion times to be *dimeric* (Fig 2C). Both of these methods (see Methods section), which—importantly—are shape independent, return a dimer-associated value for the number of Alexa fluorophores per particle: the photon counting histogram (PCH) method gave a value of 2.33, and the FCS autocorrelation curve method a value of 2.21. Fourth, elimination of the HTT^NT^ segment leads to loss of multimerization, as expected if dimer and tetramer formation are primarily driven by the HTT^NT^ segment [15,16,18,37]. Thus, a 150 nM PBS solution of Q_37_ peptide containing the P_10_ segment but lacking the HTT^NT^ segment (Fig 1C) yields diffusion times associated with monomer as the single component, instead of the tetramer observed for 150 nM HTT^NT^Q_37_P_10_C*K_2_ (Table 1). The above data strongly implicate a regular multimerization process as being responsible for observed diffusion times.

### HTT exon1 analogs in simple buffer: repeat-length and time-dependent aggregation

Further incubation of the Q_23_ molecule for 90 mins before FCS data were collected produced no change in the data (Fig 2A). However, 90 mins pre-incubation of the Q_37_ molecule generated a much more complex pattern featuring a series of spikes of high fluorescence intensity in addition to the narrow intensity fluctuations seen before (Fig 2A). When these data are analyzed by a two-component fit, they give a good correlation with a model consisting of tetramers plus a collection of larger, diffusible oligomers averaging 84 molecules of HTT exon1 per aggregate (Table 1). Interestingly, incubation of a substantially higher concentration of K_2_Q_37_P_10_C*K_2_ lacking HTT^NT^ does not lead to formation of any detectable diffusible oligomers even after 90 mins (Table 1), supporting earlier studies suggesting that HTT^NT^ is required for formation of oligomers [15,18].

To better understand the structures of these oligomers, we monitored the incubation time dependence of 2 μM solutions of HTT^NT^Q_37_P_10_K_2_ by parallel FCS and EM analysis. Two μM is the minimal concentration we have found that allows collection of meaningful EM data, and also is in the concentration range for newly synthesized HTT exon1 in our cell model (see below). By EM (Fig 2, Table 2, S1 Fig), we observed rapid formation of spherical oligomers of fairly uniform, relatively small size, ranging from an average of ~100 HTT exon1 molecules at 15 mins to an average of ~250 molecules at 30 mins (Table 2). At 60 mins EM images show a highly homogeneous array of filamentous aggregates, similar to what are generally referred to as protofibrils in other amyloid systems [46], which also contain on average ~ 700 molecules of HTT exon1 each (Table 2). At 100 mins we observed by EM a highly heterogeneous distribution of aggregates including both very large super-assemblies of filaments and very short amyloid-like fibrils; these smaller fibrils are estimated to average ~ 500 molecules of HTT exon1 per aggregate (Table 2). After 2 days of incubation we observed arrays of amyloid fibrils of uniform diameters but variable lengths. These correspond to ~ 3,000 +/- 500 molecules of HTT exon1 per fibril (Table 2). Previously, similar spherical oligomers, protofibrils and short fibrils have been reported for HTT fragments aggregating *in vitro* and *in vivo* [18,30,34,37]. FCS measurements showed reasonable agreement with the rough estimates of particle size based on analysis of EM images (Table 2). At all reaction times examined, the data were well described by a two-component fit in which one component is a tetramer and the other is a collection of larger oligomeric structures whose sizes increase as reaction time increases. At 20–30 mins the larger component has an average size of 250–350 molecules per particle; at 60 mins, ~300 molecules per particle; and at 90 mins, ~850 molecules per particle. By 120 mins diffusion times give a value of about 2,600 molecules per particle, similar to the small amyloid fibrils characterized by EM after 2 days. At later times (including 2 days) reaction time points give FCS data that cannot be fit to a model, as normally occurs with larger, heterodisperse particles.

**Table 2:** Aggregation time course of HTT^NT^Q_37_P_10_C*K_2_ by EM and FCS.

	Electron Microscopy^a^	FCS	
Time, mins	# peptides^b^^,^^e^	size1, # peptides^b^	size2, #peptides^b^
5	ND^c^	4.3	-
15–20	112 ± 26	3.7	232
30	257 ± 78	3.9	369
40	ND^c^	3.6	321
60	704 ± 63	4.1	299
90–100	513 ± 86	3.5	854^d^
120	ND^c^	3.3	2,626^d^
2,880	2,964 ± 450	ND^c^^,^^d^	ND^c^^,^^d^

^a^In EM grids exhibiting both small and very large aggregates, only the small aggregates were chosen for this calculation (since the large aggregates would be invisible in FCS).

^b^Calculated number of peptide molecules per particle.

^c^ND = not determined.

^d^These samples gave very heterogeneous data sets, many of which were not analyzable by FCS

^e^All calculated sizes are averages of 10–15 aggregates.

### HTT exon1 in cells and cell extracts: repeat-length dependent multimerization and repeat length and time-dependent aggregation

**Fig 3:**
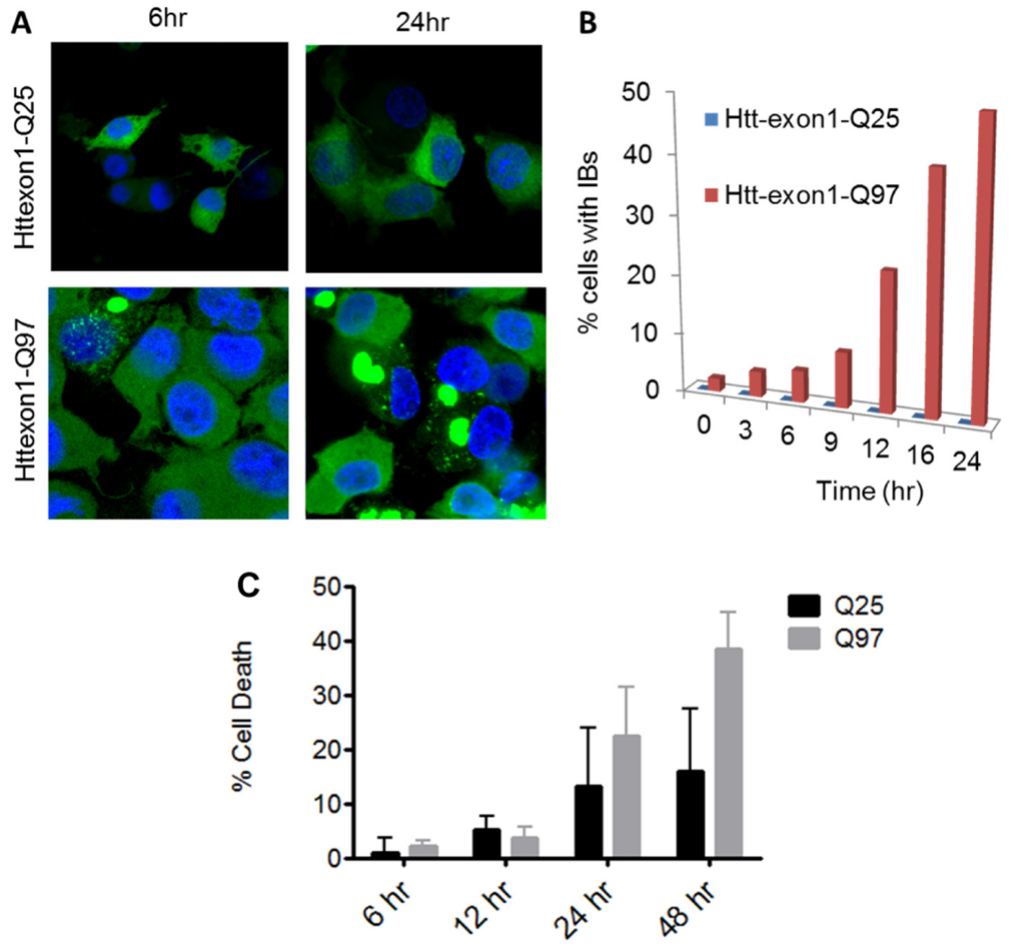
Characteristics of the PC12 model. A. Confocal microscopy of the Q25 and Q97 versions of HTT exon1 after 6 and 24 hrs growth in the presence of 1 μM ponesterone. Blue = Hoechst dye stained nucleus; green = EGFP. B. Number of cells containing inclusions at different growth times determined by fluorescence microscopy. C. Percent cell death at different growth times as determined by the amount of the intracellular enzyme lactate dehydrogenase released into the medium, an indication of the loss of outer cell membrane integrity.

We then carried out similar FCS analysis of clarified lysates of mammalian cells stably transfected and induced to produce full length HTT exon1 (Fig 1) fused at the C-terminus to EGFP and containing polyQ repeats of either 25 or 97 Gln [47]. In previous studies of this PC12 cell model, the Q_25_ version was shown to produce a diffuse EGFP fluorescence uniformly throughout the cytoplasm regardless of growth time, while the Q_97_ version exhibited diffuse fluorescence at early growth times but large inclusions at 24 hrs [47]. We observed exactly the same fluorescence microscopy behavior in our own work with these cells (Fig 3A and 3B).

**Fig 4:**
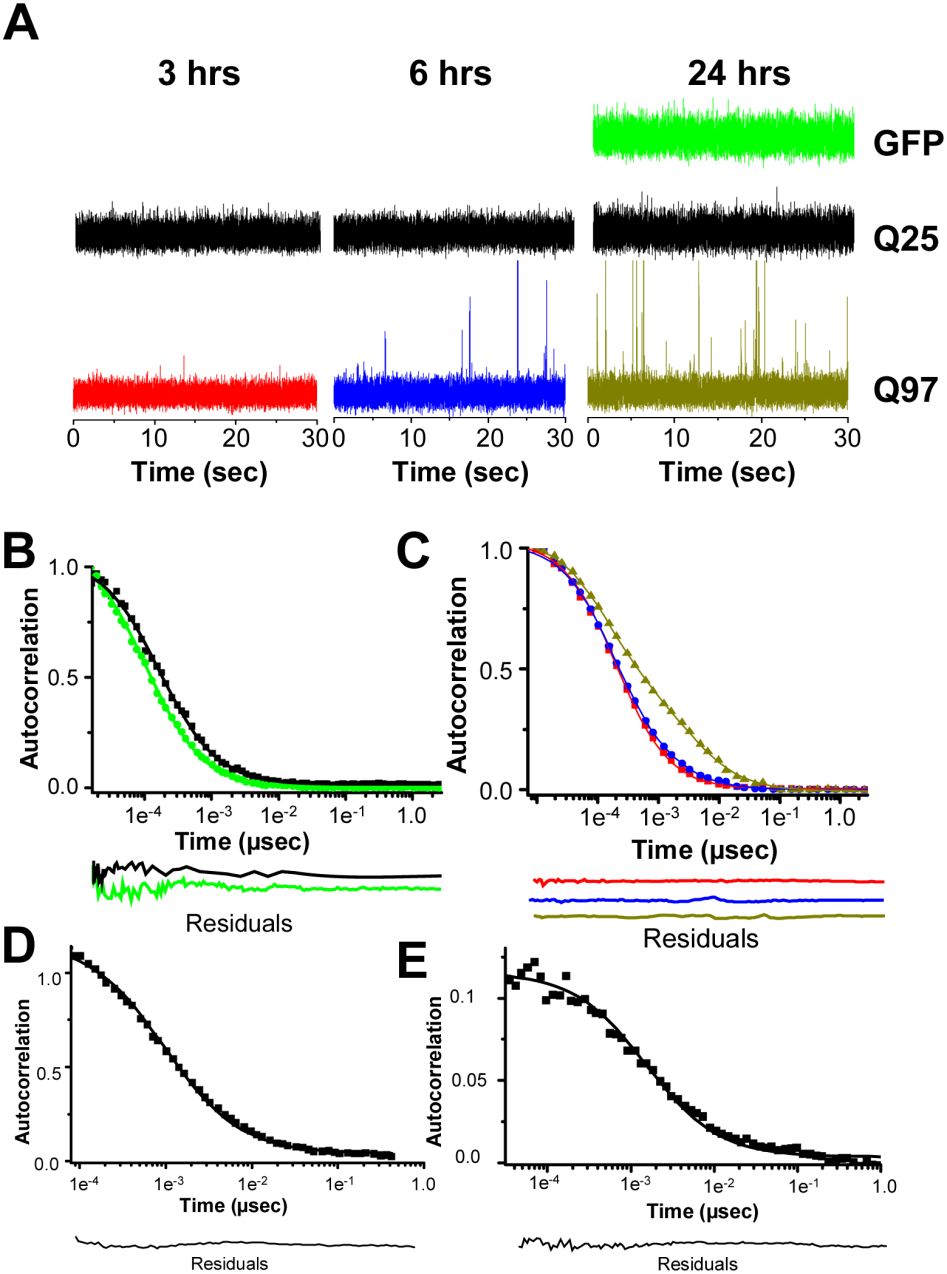
Self-assembly of full length HTT exon1-EGFP fusions in PC12 living cells and cell extracts. A. Raw FCS data from clarified native lysates of PC12 cells after different growth times. B. Autocorrelation functions with residuals of 24 hrs clarified native lysates of cells producing GFP alone (green) or HTT exon1-Q_25_-EGFP (black). C. Autocorrelation functions with residuals of native lysate supernatants of PC12 cells producing HTT exon1-Q_97_-EGFP at different growth times (color code as in part A). D, E. Autocorrelation functions with residuals of data collected from the cytoplasm of living PC12 cells producing either HTT exon1-Q_25_-EGFP (D) or HTT exon1-Q_97_-EGFP (E).

Probing further, we found that clarified extracts of Q_25_ cells grown for 3 hrs with 1 μM of the ponesterone inducer exhibit a tight range of fluorescence fluctuations (Fig 4A) giving an autocorrelation function (Fig 4B, black curve) that is best fit to a single component monomeric species (Table 1). Similar data are obtained for extracts prepared from cells grown for 6 and 24 hrs (Fig 4A), except that the FCS-evaluated monomer *concentrations* increase at longer growth times (see below). In contrast, the Q_97_ version gives data on clarified extracts of 3 hrs post-induction cells (Fig 4A) that are most consistent with a monodisperse solution of tetramers (Fig 4C, red curve; Table 1). In contrast, extracts of cells grown for 6 hrs exhibit a baseline of fluorescence attributed to tetramers, plus occasional spikes of high fluorescence intensity (Fig 4A) consistent with diffusible oligomers in the size range of 20-mers (Fig 4C, Table 1). By 24 hrs, tetramers persist, but there are also many additional fluorescence spikes (Fig 4A), the average size of which has grown to a range of ~3,000 HTT exon1 molecules per diffusible aggregate (Fig 4C, Table 1).

We also analyzed the self-association states of HTT exon1 molecules by conducting FCS in living cells. We used the confocal microscope capability of the FCS instrument to locate areas of the cell cytoplasm exhibiting diffuse green fluorescence, and then collected time-dependent fluorescence fluctuation data at these sites. We found that the EGFP fluorescence in the cytoplasm of HTT exon1-Q_25_ cells after 6 hrs exhibits an autocorrelation function consistent with a monodisperse solution of monomers (Fig 4D, Table 1), while in the HTT exon1-Q_97_ cells the fluorescence fluctuations are consistent with a monodisperse solution of tetramers (Fig 4E, Table 1). (We note that the diffusion time of GFP alone is found to be somewhat larger than that of the slightly larger EGFP-HTT exon1 fusion (Table 1); this significant but small perturbation, which must be due to an effect of the intact cellular environment on GFP or HTT exon1 since it is not observed in our *in vitro* measurements of lysates of the same cells, does not affect the main findings.)

Thus, in broad measure, our analysis of HTT exon1 producing cells gives data in total agreement with the data on HTT exon1-like peptides *in vitro*. One caveat is that it is formally possible that the fluorescent particles observed in HTT exon1-Q_97_ cells and cell extracts might be complexes of HTT exon1 with other cellular proteins, rather than multimers of HTT exon1. We think this is unlikely, however, since we observe exactly the same polyQ repeat length dependent self-association trends with chemically synthesized peptides in simple buffer as we do in cells producing HTT exon1 (Table 1). It also seems unlikely that any of the normal interaction partners of HTT exon1 would be available in sufficient amounts to saturate formation of these hypothetical complexes at the elevated concentrations of HTT exon1 (see below) resulting from the ponesterone induction system. We did not observe any fluorescent spikes in the intracellular measurements that are analogous to those seen in extracts of the same cells. This is not surprising, however, since small aggregates are expected to diffuse so slowly in the highly viscous cytoplasm that their diffusion times cannot be assessed by FCS. It is also possible that such aggregates would not freely diffuse at all, by virtue of their interactions with cellular structures, such as those tasked with eradicating protein aggregates [48].

### HTT exon1 concentrations and molecular flux in growing cells

**Fig 5:**
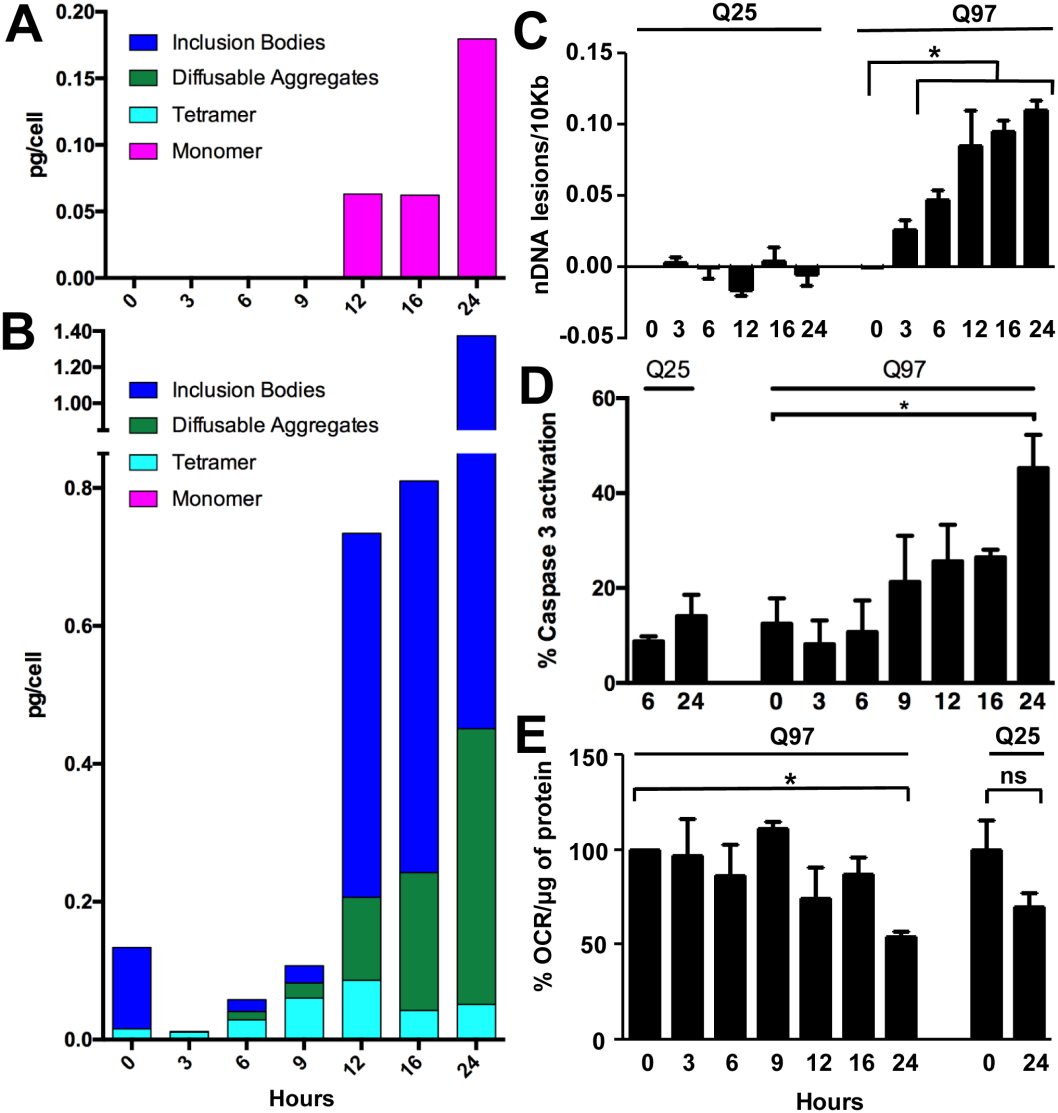
Time courses of PC12 cell responses to HTT exon1 expression. A, B. Cell levels of monomer, tetramer, diffusible aggregates and inclusions in cells producing HTT exon1-Q_25_-EGFP (A) or HTT exon1-Q_97_-EGFP (B) analyzed at different times after onset of growth with inducer. In A, FCS measurements yielding information on monomers, dimers/tetramers, and diffusible oligomers were made 12, 16 and 24 hrs. In B, except for the lack of a measurement for inclusions at 3 hrs, absence of a value for a species indicates a measurement yielding a value of zero. C, D, E. Growth time dependent effects of expression of HTT exon1 with a Q_25_ or Q_97_ repeat on nuclear DNA damage (C), caspase-3 activation (D) and cell mitochondrial respiration (E). OCR = oxygen consumption rate.

Autocorrelation functions also yield information on the number of particles in the focal volume [40,41], and from this information we can obtain estimates of the cellular concentrations of different species (Materials and Methods). We took advantage of this to construct a detailed time course of the cellular production of various HTT exon1 species (Fig 5). To avoid confusion caused by changing molecular weights of different multimers, we plot the results in Fig 5 on a mass/volume basis rather than a moles/volume basis. We found that the cellular content of HTT exon1-Q_25_ monomers rises to about 0.18 pg/cell (approximately 18 μM) after 24 hrs growth post induction, while no tetramers or diffusible aggregates are detected at any time (Fig 5A). In contrast, for HTT exon1-Q_97_, no monomers are detected at any time (Fig 5B). Instead, tetramers exist from the earliest measurement time and exhibit a biphasic response to growth time, growing to a maximum of ~ 0.08 pg/cell (equivalent to a cellular concentration of about 8 μM of HTT exon1 molecules incorporated into tetramers) at 12 hrs, and then decaying to a value of ~ 0.05 pg/cell (or about 5 μM of total HTT exon1 molecules) at 24 hrs (Fig 5B). Larger, diffusible aggregates emerge after 6 hrs of growth and continue to rise to a final value of 0.4 pg/cell at 24 hrs (Fig 5B). It is not possible to detect larger aggregates, such as inclusions, by FCS, since these do not freely diffuse even in lysates. Therefore, to get a complete picture of the partitioning and flux of cellular HTT exon1-Q_97_, we used Western blot analysis (Methods) to determine the amounts of HTT exon1 found in the pellets of the clarified cell lysates, a pool expected to contain the remaining aggregated HTT exon1 material consisting of larger amyloid fibrils and inclusions. We found that while such sedimentable material represents a very small amount of HTT exon1 in the first 9 hrs of growth, levels of this class of HTT exon1-Q_97_ material rise dramatically at 12 hrs and continue to rise through 24 hrs (Fig 5B) to a nominal concentration of over 100 μM.

### Time dependent development of cell dysfunction and death

As previously reported for this PC12 cell model [28,47], cells making HTT exon1 with either a Q_25_ or Q_97_ polyQ repeat undergo growth time dependent cell death about equally over the first 24 hrs, after which continued cell death is more pronounced for the HTT exon1-Q_97_ cells (Fig 3C). Although our primary interest in this work is to use the cell model to study repeat length dependent self-association of HTT exon1, we took advantage of the HTT exon1-dependent cell death phenotype to study the timing of appearance of cell dysfunction and death as it compares to the timing of aggregation phenomena. We analyzed time dependent development of nuclear DNA damage [49,50], caspase 3 activation [51], and deficiencies in mitochondrial respiration [52]. In a particularly striking result, we observed almost immediate nuclear DNA damage in the cells expressing HTT exon1-Q_97_, with the value rising to significance by 6 hrs and continuing to grow over the first 24 hrs post-induction (Fig 5C). In contrast, we found no evidence for nuclear DNA damage over this 24 hrs period in the HTT exon1-Q_25_ cells (Fig 5C). Likewise, we observed caspase 3 activation to increase slowly in the HTT exon1-Q_97_ cells (but not Q_25_ cells), reaching significance at 24 hrs (Fig 5D). Finally, we recorded oxygen consumption rates in living cells over a 24 hr period post-induction, normalizing the mitochondrial viability data to the number of living cells at each time point (Materials and Methods). We observed a decline in oxygen consumption rate (OCR) at 24 hrs in cells expressing HTT exon1-Q_25_, but this did not reach statistical significance (Fig 5E). Cell respiration declined steadily in the Q_97_ cell culture, finally reaching significance at 24 hrs (Fig 5E). To investigate whether this decay in respiratory efficiency was due to a change in status of the mitochondria or to a change in the metabolic pool, we permeabilized HTT exon1-Q_97_ cells at 24 hrs, supplied a buffer complete with required substrates, and monitored respiration (S1 File). The results (S2 Fig) continue to show a HTT exon1-expression dependent decay in respiration, suggesting that the data in Fig 5F owe to a reduction in fully capable mitochondria. Thus, several indicators of cell stress develop over the first 24 hrs after expanded polyQ HTT exon1 expression is initiated and unique self-associated forms begin to accumulate.

## Discussion

We describe here robust FCS data showing several molecular states of expanded polyQ repeat HTT exon1 that are not formed by normal repeat length versions. Short polyQ versions tend to exist in the monomeric state, forming dimers at higher concentrations, but resist forming higher aggregates. Expanded polyQ versions populate a dimer to tetramer equilibrium, with no evidence for the existence of a monomeric form even at concentrations as low as 300 pM (Fig 2C). One immediate consequence of our results is the need to refine our model for HTT exon1 self-association (Fig 1) to include the presence of dimeric forms which were not observed in previous AUC experiments on simpler HTT^NT^ constructs [15]. The general trends in self-association observed here are seen with chemically synthesized peptides in PBS as well as with recombinantly expressed peptides in cell extracts and living cells, and are also very similar whether the FCS probe is a small molecule or a fluorescent protein.

Previously we noted that both the formation of non-β oligomers from monomer and the nucleation of amyloid fibril formation from within these oligomers appear to independently exhibit polyQ repeat length dependence [15,18]. Our new data demonstrate that this repeat length dependence extends to the earliest observed self-association events. Thus, HTT^NT^Q_23_P_10_C*K_2_ populates dimers at 40 nM up to nearly 1 μM, while HTT^NT^Q_37_P_10_C*K_2_ populates dimers in the range of 300 pM up to 3 nM (Fig 2C). A limiting value for the magnitude of this stabilization effect can be estimated from the curves in Fig 2C that suggest that the midpoints of the monomer to dimer concentration dependent curves for these two peptides must be separated by at least 100-fold. This sizable stabilizing effect is attributable to a difference in only 14 Gln residues. We can understand this effect in the context of a leading role for HTT^NT^ in mediating homotypic multimer formation, as suggested previously [15,16,18,53] and demonstrated here by the loss of dimer formation, even at relatively high concentrations, when HTT^NT^ is removed from an HTT exon1-like peptide (Table 1, Q_30_ series). Thus, while computationally predicted [54] homotypic polyQ interactions appear to be too weak to produce multimers of an HTT exon1 analog lacking the HTT^NT^ domain (Table 1), they appear to be sufficiently strong to enhance self-association mediated by HTT^NT^-HTT^NT^ interactions.

It is not immediately obvious why FCS should reveal an HTT^NT^-mediated multimerization that is sometimes not detected by other methods such as AUC [14] and SEC [10]. Although SEC is normally considered to be a robust measurement, it nonetheless involves shear forces and matrix surface chemistries that could potentially introduce artefacts. We note that while some HTT^NT^-containing peptides behave as multimers by AUC, they migrate as monomers in SEC [15]. In addition, the chemical state of the HTT^NT^ segment may play an appreciable role in multimer stability, as recently suggested by ion-mobility mass spectrometry analysis [16]. In this context it may be relevant that most [9,14,26,30,55], but not all [56], cleavable fusion protein systems for recombinant expression of HTT exon1 generate N-termini that are modified from the gene-encoded Met-Ala-Thr start used in our chemically synthesized and directly expressed constructs. It appears that α-helical multimeric forms of intrinsically disordered proteins are generally relatively fragile. For example, observation of helical tetramer formation by α-synuclein [57] depends on the chemical state of the N-terminus, on isolation conditions, and on the method of analysis [58].

Expanded polyQ versions also exhibit time dependent formation of several types of higher aggregates. *In vitro* with 2 μM HTT^NT^Q_37_P_10_K_2_, we observed formation within 15–30 mins of spherical oligomers containing 50–100 molecules of HTT exon1, then protofibrils and small, diffusible fibrils containing hundreds of molecules of HTT exon1 at about 60 mins, then short, diffusible amyloid fibrils in the range of 3,000 molecules of HTT exon1 by 120 mins (Fig 2D, Table 2). Importantly, diffusible HTT exon1 particles in this same 2,000–3,000 size range begin to accumulate in PC12 cells in the 12–24 hrs range (Table 1); the similarly of their FCS-determined sizes suggests that these cell-produced aggregates are diffusible amyloid fibrils similar to those observed during *in vitro* self-association. We expect that the somewhat smaller protofibrillar structures identified in the *in vitro* experiments (Fig 2D; S1 Fig) are also transiently formed in the cells; in other amyloid systems, such protofibrils exhibit significant β-structure [59]. Overall, our data show that formation of initial diffusible aggregates is very efficient, taking place over only a few minutes *in vitro* (Fig 2; Table 2) and a few hours in cells (Table 1). The very fast time course of diffusible aggregate formation even at low concentrations *in vitro* (Fig 2; Table 1) illustrates the severe challenges facing investigators who aim to investigate the *in vitro* physical and biological properties of low MW forms of HTT exon1-like peptides.

The last assembly state to form in the cell environment is the collection of very large aggregates, including isolated clusters of long amyloid fibrils as well as inclusions, that pellet in a low-speed centrifugation and are only observable by their presence in cell lysis pellets or by fluorescence [28] or super-resolution fluorescence [38] microscopy of fixed cells. According to our combined FCS and Western blot data, formation of aggregates clearly suppresses protein turnover and provides a huge net enhancement of total accumulated HTT exon1 levels in the cell, so that while the total concentration of HTT exon1-Q_25_ in the cell at 24 hrs is about 2 μM (Fig 5A), the total nominal cellular concentration of the HTT exon1-Q_97_ peptide in all physical forms at 24 hrs (Fig 5B) is about 150 μM! Our results are also consistent with previous reports [32,39] showing that concentrations of low MW forms of expanded polyQ HTT exon1 exhibit a biphasic response to growth time due to the late development of large amyloid aggregates acting as thermodynamic sinks that deplete low MW species as they are synthesized. Thus, tetramers of HTT exon1-Q_97_ reach a maximal cellular concentration at 12 hrs, then—in spite of continued protein production at the ribosome—begin to decline as diffusible aggregates and inclusions build up at later time points (Fig 5B).

The sensitivity of FCS reveals the formation of diffusible aggregates within 20 mins for a 2 μM PBS solution of HTT^NT^Q_37_P_10_K_2_ (Table 2) and within 90 mins for a 30 nM solution (Table 1). In contrast, when the substantially longer Q_97_ form of HTT exon1 is expressed at low μM levels in the cell, it requires 6–9 hrs before very small diffusible aggregates begin to be observed (Table 1). There are a number of possible explanations for why aggregation is dramatically slowed in the cell, in spite of conditions in the cell experiments (much longer polyQ repeat, micromolar concentrations, and molecular crowding) that would normally be considered to favor aggregation. For example, some cellular post-translational modifications within the critical HTT^NT^ segment have been shown or postulated to directly slow aggregation [3,60,61]. In addition, the intrinsically disordered HTT^NT^ segment is predicted to be a molecular recognition feature (MoRF) [62] capable of coupled folding and binding to other proteins [5,18], and it is clear that the aggregation enhancing effects of HTT^NT^ are largely neutralized when it is bound to other proteins such as some molecular chaperones [53,63]. Thus, while HTT exon1 aggregation in cell models appears to be quite robust, it nonetheless is actually considerably suppressed compared with aggregation rates observed in simple buffer systems. Whatever the detailed mechanisms of suppression, it is possible that modest variations in the efficiency of these mechanisms might help explain variable susceptibilities to particular polyQ repeat lengths by neuronal cell types and by HD patients [64].

Our linked EM and FCS data suggest that very small amyloid fibrils and protofibrils with lengths in the range of 50–100 nm are part of a general class of diffusible (often referred to as “soluble”) aggregates forming very early in expanded polyQ HTT exon1-producing cells, where they constitute a bridge in the timeline between spherical, non-β oligomers and large amyloid fibrils and inclusions. Such a result contrasts with recent super-resolution fluorescence microscopy studies which showed that recognizable amyloid fibrils, with lengths up to 1.5 μm, only begin to form in HTT exon1 producing cells *after* the appearance of inclusions [35]. However, the resolution of super-resolution methods may be insufficient to distinguish between fibrils and spherical oligomers in particles in the sub-μm size range; in contrast, we observe fibrils and protofibrils in the EM with lengths in the 50–100 nm range (Fig 2D). Alternatively, our inference, based on these *in vitro* EM studies, that diffusible particles containing 2,000–3,000 molecules found in cell extracts are in fact fibrillary versions of HTT exon1 may be mistaken. Resolving this issue will be important, since it may help us narrow or expand the range of physical states of HTT exon1 that exist during the earliest stages of the cytotoxic response. Diffusible amyloid fibrils are an exciting addition to the list of potentially cytotoxic forms of aggregated HTT fragments, since they would be capable of recruiting other cellular polyQ proteins through amyloid elongation in the cell [65], a process previously suggested as a possible source of cytotoxicity [27,66].

Although our focus has been on cellular self-assembly of HTT exon1, time-dependent cell dysfunction (Fig 5C–5E) and cell death (Fig 3C) measurements under the same growth conditions provide and interesting context for the aggregation data. Our measurements of the temporal appearance of various types of cell abnormalities provide confirmation in the PC12 model that mitochondrial dysfunction [52,67] and caspase activation [51] are associated with expanded polyQ HTT exon1 cytotoxicity. Statistically significant levels of abnormalities begin to appear at 24 hrs post-induction, long after all the various self-associated forms of HTT exon1-Q_97_ described here have made an appearance. Thus, by these measures, any one of these species shown to develop over this time frame in expanded polyQ HTT exon1 cells but not in WT HTT exon1 cells might be responsible for triggering toxic events. However, we also find strong evidence for the time-dependent, early development of nuclear DNA damage in the expanded polyQ HTT exon1 cells, with statistically significant levels of DNA damage already begin to appear by 6 hrs. At this time monomers are undetectable and the steep development of large fibrils and inclusions has not yet begun (Fig 5B). Instead, the HTT exon1-Q_97_ protein is distributed between tetramers and diffusible aggregates that likely consist of both non-β oligomers and small protofibrils and fibrils (Fig 5B). If the striking DNA damage can be considered to an early aspect of expanded polyQ toxicity, from which later-developing cellular dysfunctions develop, the data suggest that toxicity might be triggered by HTT exon1 tetramers, non-β oligomers, and/or small amyloid fibrils. The lack of precision in this conclusion illustrates the difficulties in assigning toxicity to a specific form of HTT exon1, since the on-going aggregation process, fueled by new production of the peptide at the ribosome and quickly transiting through multiple intermediates, populates a number of forms even at the earliest stages. In addition, it should be pointed out that since we don’t know the steady state concentration of low MW forms of HTT exon1 in neurons, nor do we know the midpoint concentration for the monomer-dimer equilibrium for expanded polyQ HTT exon1 (see Fig 2C), it remains possible that monomers of expanded polyQ HTT exon1 might be at least partially populated at normal cellular concentrations. Future studies investigating both of these open issues are potentially critically important to the understanding of HD disease mechanisms.

## Materials and Methods

### Materials and General Methods

Synthetic peptides were obtained as crude products from the small scale peptide synthesis facility of the Keck Biotechnology Center at Yale University. Peptides were purified by reverse phase HPLC, reacted with AlexaFluor-555 C2 maleimide using the protocol of the supplier (Life Technologies, #A20346), and repurified by HPLC [68]. Host PC12 cells (“Schweitzer morph A” subclone) and stably transfected derivatives of these cells carrying genes for the Q_25_ and Q_97_ versions of HTT exon1 C-terminally fused to EGFP [47] were obtained from Erik Schweitzer. Host PC12 cells were transiently transfected to produce GFP alone by nucleofection using Lonza Kit V per the manufacturer’s optimization protocol for PC12 cells.

### Htt-exon1 expression and cell lysate preparation

PC12 cells were maintained in Dulbecco’s modified media (DMEM) containing 25 nM HEPES (Cellgro), 5% supplemented calf serum (Hyclone), 5% horse serum (Hyclone), 2 mM l-glutamine, 0.5 mg/ml G418 (Mediatech and cultured on collagen IV coated plates (Trevigen) at 37°C in 9.5% CO2 ([47] and Erik Schweitzer, personal communication). Cells were induced with 1 μM ponasterone (Invitrogen) and collected by scraping at different time points after induction. Cells were washed in warm PBS and pellets were stored at -20°C until analyzed. Frozen cell pellets were thawed on ice and resuspended in non-denaturing lysis buffer (50 mM Tris-HCl, pH 7.4, 150 mM NaCl, 0.5% v/v Triton X-100) with added protease inhibitor cocktail (Sigma, P8340-1ML). Samples were cleared by 30 min centrifugation at 2000 g at 4°C and kept on ice until analyzed.

### Fluorescence Correlation Spectroscopy

#### General

All FCS experiments were performed on a Carl Zeiss LSM-510 META MP ConfoCor 3 at the FCS facility at Carnegie Mellon University according to described general procedures [40]. Measurements of Alexa fluor 555 labelled peptides were carried out with a He-Ne laser (561 nm line) and of EGFP and GFP with an Ar laser (488 nm line). Care was taken to avoid exceeding the photon counting limit of the detectors. In peptide experiments, this entailed mixing labelled with unlabeled peptide when concentrations higher than about 0.5 μM were required. For cell lysate experiments, this entailed carrying out an appropriate dilution of the clarified lysate in the non-denaturing lysis buffer (above). For live cell experiments, this entailed keeping post-induction growth times to 6 hrs or less, and/or identifying specific target cells with appropriate fluorescence intensities.

#### Peptides

Prior to peptide measurements, cover slides were pretreated with 1 mg/ml BSA solution for 15 mins then rinsed with water; this pretreatment was required to suppress adsorptive loss of peptides from solution at low peptide concentrations. Alexa-tagged peptides were disaggregated and their concentrations determined and adjusted [68], and pH 3 stock solutions were aliquoted into vials, snap frozen in liquid nitrogen, and stored at -80°C prior to use (a control experiment was performed to ensure that frozen storage over a limited time did not alter results). For FCS, aliquots of the thawed stock solution were adjusted in volume to attain the desired concentration in PBS buffer. Samples aliquoted onto the BSA-treated slides were then incubated for 10 mins (or longer, as indicated) before collecting data.

#### Live cells

Cells were plated on glass coverslips in Petri dishes, transferred to the FCS facility, and acclimated in an appropriate incubator for at least one day prior to the experiment.

#### Cell lysates

Clarified cell lysates were prepared as described above, transferred on ice to the FCS facility, and diluted into non-denaturing lysis buffer and analyzed.

#### Analysis

The temporal auto-correlation containing information about concentration and diffusion time of the fluorescent molecules is given by $G\left(\tau \right)\;=\;\sum {}_{i}n_{i}\left(\frac{1}{1+\tau_{Di}}\right)\left(\frac{1}{1+a^{2}\tau_{Di}}\right)$, where *i* is the number of components and *τ*_*D*_ is the diffusion time [40]. All FCS data were fit to either one component or two components depending on the goodness of the fit. The information about the relative number of particles of each type is calculated from the coefficients *n*_*i*_. For a single component analysis, the concentrations were calculated directly from the *G(0)* which is *1/N*, *N* being the number of particles in the focal volume. For two component fits, $n_{i}\;=\;c_{i}\emptyset_{i}^{2}$. The focal volume was calculated using either GFP or Rhodamine B. Knowing the number of particles and the focal volume, the molar concentration can be calculated. This was converted to picogram/cell using the calculated molecular weight of the particle of interest, knowledge of the dilution series applied to the sample, and a standard curve relating cellular protein mass to cell number (S2 Fig, panel B; see below). All data fitting was done in Origin 7.5.

Brightness analysis, under conditions where the autocorrelation analysis showed only one species, was carried out by two methods. For the autocorrelation curve method, the number of particles in the observation volume was calculated from the autocorrelation function and the relationship G(0) = 1/N. Brightness was calculated by taking the ratio of fluorescent count rate and N. We compared the brightness of the labelled HTT exon1 solution with that of a solution of Alexa dye, to generate a ratio equivalent to the number of molecules per particle [40]. For the photon counting histogram (PCH) method, we analyzed stored raw counts output using the PCH analysis plugin software for ImageJ software (http://imagej.nih.gov/ij/) provided by Jay Unruh of the Stowers Institute for Medical Research in Kansas City, MO as described elsewhere [45]. The analysis yields a histogram distribution of fluorescence intensity fluctuations, the average number of molecules in the focal volume, and their molecular brightness.

### Electron microscopy imaging and estimating monomer content of particles

Aliquots of some reaction mixtures were taken at various time points and analyzed immediately, without further manipulations, by electron microscopy. EM was carried out as described [69]. The number of molecules in oligomers, protofibrils, and fibrils imaged in the EM analysis (Table 2) was approximated by: (a) determining the dimensions of each particle using ImageJ software (http://imagej.nih.gov/ij/), (b) calculating the internal volumes of each (treating oligomers as spheres and protofibrils/fibrils as cylinders), (c) estimating the mass of the particle using a value for the density of a folded, globular protein of 1.37 g/cm^3^ [70],and (d) calculating the number of molecules of peptide in the particle using its molecular weight and Avogadro’s number. Approximate particle sizes for each class were then determined by averaging 10–15 example species of each type.

### Standard curve of the number of cells versus protein

In order to put analytical HTT exon1 data on a per cell basis over the course of a cell growth experiment, we developed a correspondence between cell number and total protein. PC12 cells were grown in normal media until ~70% confluent, trypsinized, pelleted, and counted using a hemocytomer. Cell counts ranging from 10,000 to 400,000 were aliquoted and lysed using 100 μL of RIPA buffer (Sigma, R0278-50ML). The protein concentration was measured using a standard Bradford assay. The resulting standard curve (S2 Fig, panel B) was used to determine the number of cells represented in the lysates analyzed by FCS and by Western blot quantification of inclusions.

### Estimating cellular HTT exon1 levels by GFP Western blot

Levels of soluble (monomers, tetramers, and diffusible aggregates) and insoluble (sedimentable amyloid and inclusions) forms of HTT exon1-Q_97_ were determined using a GFP epitope in SDS-PAGE Western blots. See S1 File.

### Caspase 3 activation analysis

Aliquots of clarified lysates (enough volume to deliver 20 μg total protein) of cells harvested at different times were run on SDS PAGE gels and probed with a rabbit antibody against cleaved caspase-3 (Asp175, Cell Signalling, #9661S) for WB determination of active phosphorylated caspase-3. Values were normalized to the level of caspase-3 activation in host PC12 cells grown for 9 hrs in media supplemented with 5 μM staurosporine (Promega, # A8192).

### DNA damage

Nuclear DNA damage was assessed as described [49] and modified [50]. The assay detects several types of damage including strand breaks, abasic sites, repair intermediates and others.

### Cell respiration analysis

HTT exon1-Q_25_ or HTT exon1-Q_97_ PC12 cells per well were seeded onto a 24XF plate and induced with 1 μM ponasterone at the required time points. The day of the experiment plates were checked for even confluency and 1 hour before the measurement cells were incubated with XF assay media (Seahorse Bioscience cat# 102365–100, 1mM sodium pyruvate, 4.5 g/L Glucose) at 37C without CO_2_. XF sensor cartridges were hydrated overnight at 37°C, no CO_2_, in XF Calibration Buffer cat# 100840–000) and one hour before the measurements the cartridge was loaded and calibrated in the XF24 instrument. Basal OCR (oxygen consumption rate) was recorded using a Seahorse XF24 Extracellular Flux Analyzer for at least 1 hour. To normalize the OCR results to the amount of cells present in each well, the media was removed after the measurements and cells were lysed in RIPA buffer and a BioRad protein assay (cat# 500-0113/4) was carried out to quantify micrograms of protein per well. Methods of assessing the respiration of permeabilized cells are described in S1 File.

### Cytotoxicity

As a measure of cytotoxicity the release of lactate dehydrogenase (LDH) into the growth medium by cells with damaged membrane was quantified using the CytoTox-ONE^™^ Homogeneous Membrane Integrity Assay (Promega) in 96-well plate format.

### Statistical analysis

Cellular data were analyzed by GraphPad PRISM. Significance was determined using one-way ANOVA, followed by post-hoc analysis (Student's t-tests with Bonferroni correction) using p < 0.05.

## Supporting Information

S1 Fig Time-dependent morphologies by EM of a 2 μM solution of HTT^NT^Q_37_P_10_K_2_. (PDF)

S2 Fig Details of cell measurements. (PDF)

S1 File Additional Materials and Methods. (PDF)
