# Supplementary material for: Folding Landscape of Mutant Huntingtin Exon1: Diffusible Multimers, Oligomers and Fibrils, and No Detectable Monomer
Source: PLoS One. 2016 Jun 6;11(6):e0155747. doi: 10.1371/journal.pone.0155747 (PMC4894636; doi:10.1371/journal.pone.0155747)
Supplement: S1 Fig — (PDF) [file pone.0155747.s001.pdf]

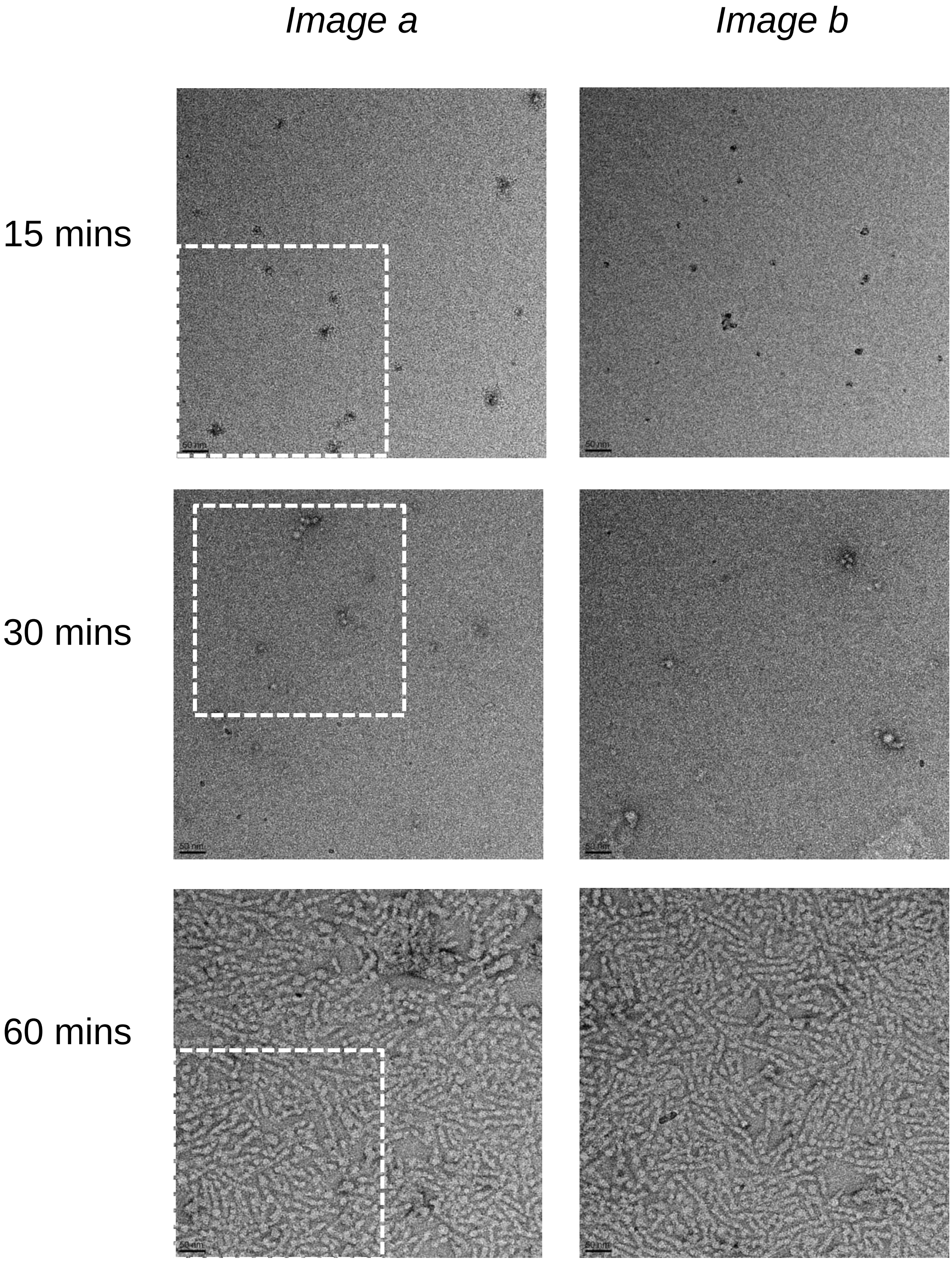
Image a
Image b
15 mins
30 mins
60 mins

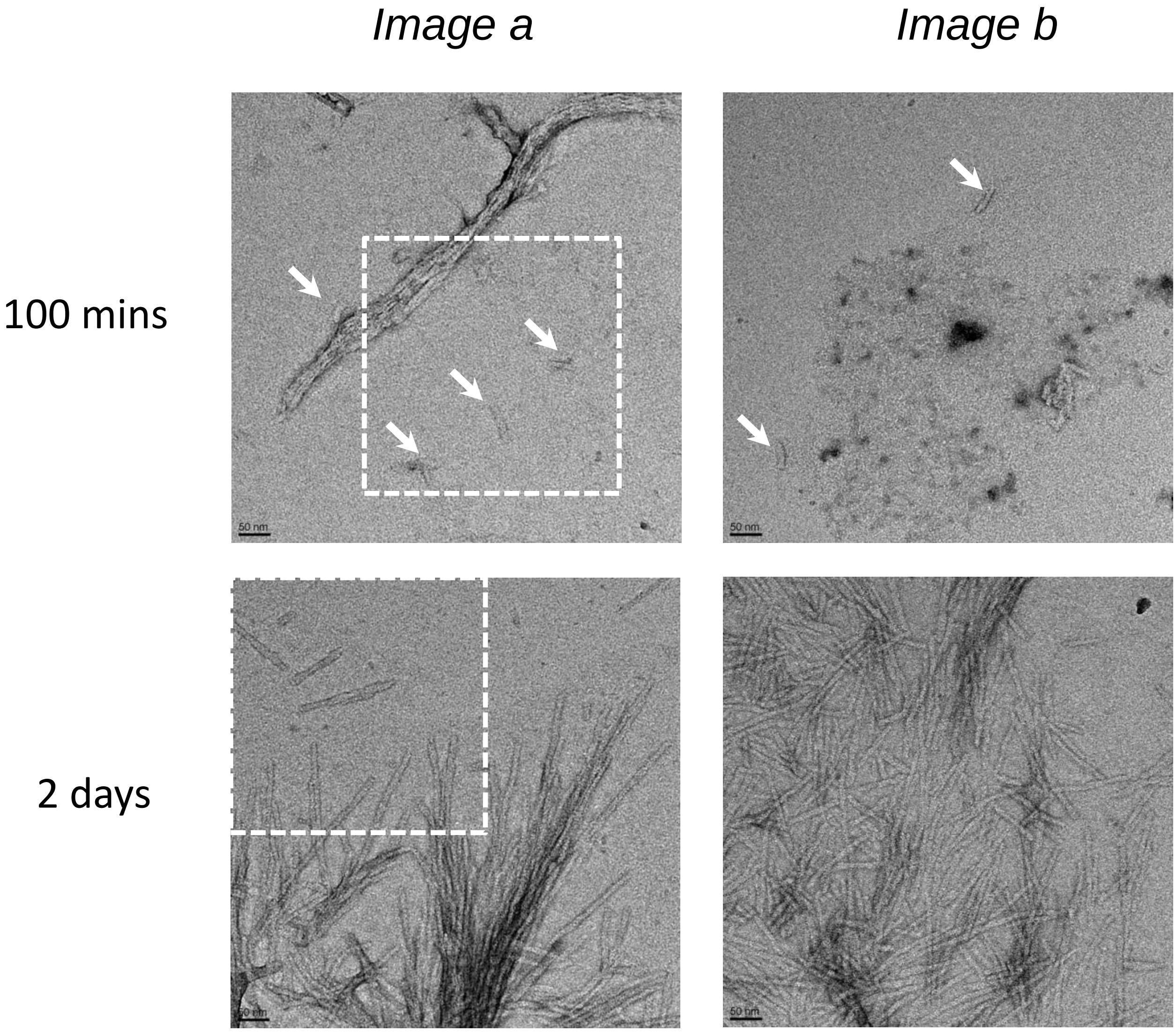

S1 Figure. Time-dependent morphologies by EM of a 2 µM solution of $HTT^{NT}Q_{37}P_{10}K_2$. EM images of different time points in the incubation of a 2 µM solution of $HTT^{NT}Q_{37}P_{10}K_2$ in PBS at 37 °C. Two EM grids are shown for each time point, illustrating the degree of uniformity of the reactions at different times. The white dashed boxes correspond to the portions reproduced in Figure 2. At 15 mins, aggregates are spherical and positively staining. At 30 mins, aggregates are spherical and negatively staining. At 60 mins, aggregates appear to be negatively stained strings of the spherical oligomers appearing at 15 mins, a form often referred to in the amyloid literature as "protofibrils". At 100 mins the reaction mixture is much more heterogeneous, consisting of negatively stained bundles of long, thin filaments (image a) and other large assemblies, surrounded by very short, negatively stained fibrils (arrows). After two days, long, negatively stained fibrils have developed.
