# Supplementary material for: Folding Landscape of Mutant Huntingtin Exon1: Diffusible Multimers, Oligomers and Fibrils, and No Detectable Monomer
Source: PLoS One. 2016 Jun 6;11(6):e0155747. doi: 10.1371/journal.pone.0155747 (PMC4894636; doi:10.1371/journal.pone.0155747)
Supplement: S2 Fig — (PDF) [file pone.0155747.s002.pdf]

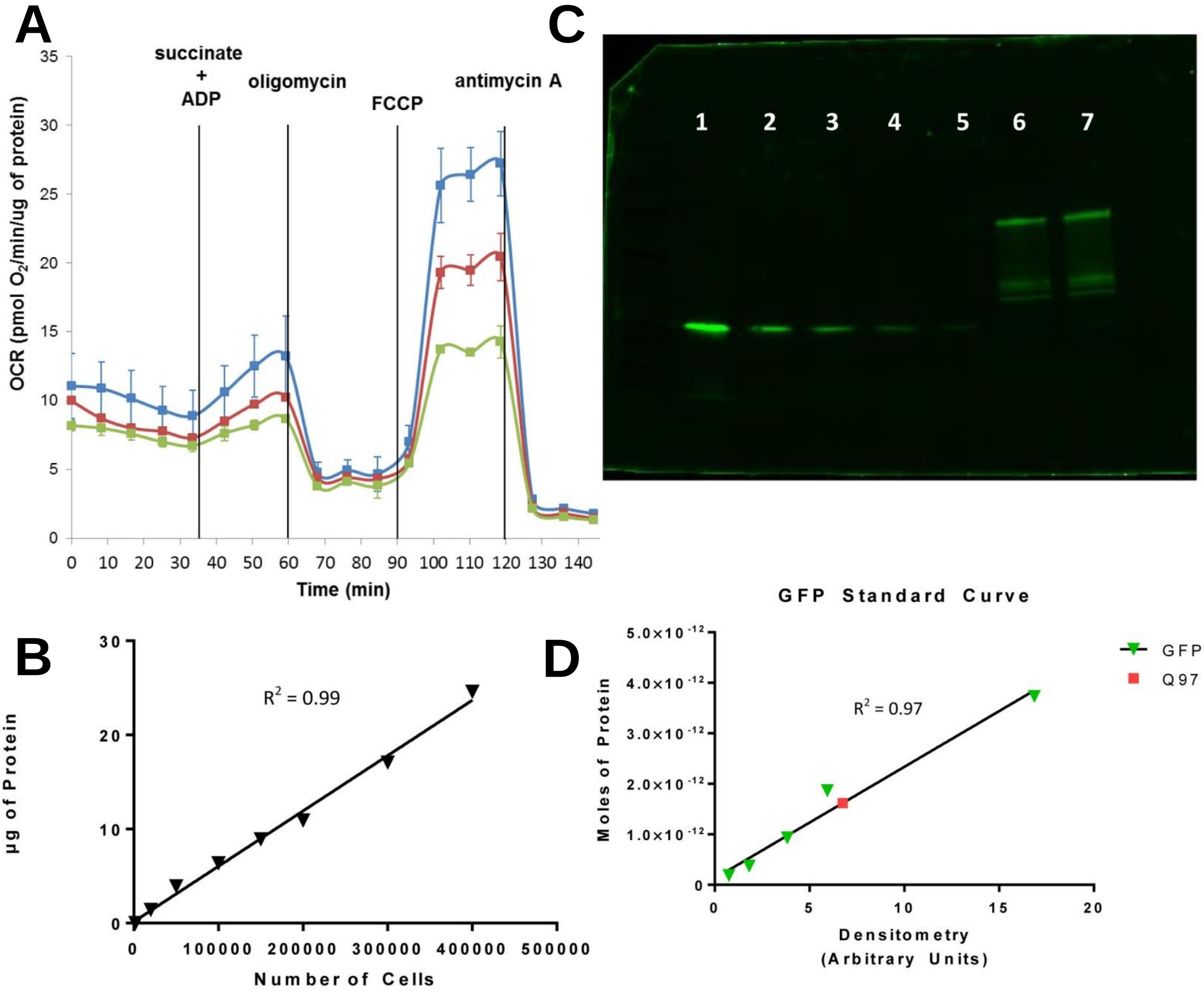

S2 Figure. Details of cell measurements. (A) Effect of small molecule metabolites on cell respiratory activity (S1. Supporting Information). After 24 hrs growth in the presence of ponasterone inducer (0 µM, blue; 0.5 µM, red; 1.0 µM, green), cells were permeabilized with digitonin for 10 minutes in the presence of a cocktail of mitochondrial respiration substrates and OCR measured either with no further treatment (State III of respiration), with added succinate (State IV) or after FCCP treatment (uncoupled state). All three respiration states are inhibited, suggesting that decreased mitochondrial respiration in HTT-exon1-Q_{97} expressing cells is due to direct electron transport chain damage. (B) Standard curve of the number of cells versus µg of total protein extract in lysed PC12 cells used to determine the number of PC12 cells represented in cell lysates analyzed for various forms of HTT exon1 (Materials and Methods). (C, D) Standard curve determination of HTT exon1 mass in a cell extract. (C) Example of a concentration determination of HTT-exon1 in cell extracts by SDS PAGE Western blot (Lane 1 = 0.1 µg GFP, Lane 2 = 0.05 µg GFP, Lane 3 = 0.025 µg GFP, Lane 4 = 0.01 µg GFP, Lane 5 = 0.005 µg GFP, Lanes 6 and 7, duplicate loadings of an aliquot of resuspended cell lysis pellet from HTT exon1-Q_{97} cells grown 12 hrs). (D) Standard curve and example analyte from densitometry of the gel shown in C, showing the standards (▼) and the HTT exon1-Q_{97}, 12 hrs (■) densities (S1. Supporting Information).
