# Supplementary material for: Folding Landscape of Mutant Huntingtin Exon1: Diffusible Multimers, Oligomers and Fibrils, and No Detectable Monomer
Source: PLoS One. 2016 Jun 6;11(6):e0155747. doi: 10.1371/journal.pone.0155747 (PMC4894636; doi:10.1371/journal.pone.0155747)
Supplement: S1 File — (PDF) [file pone.0155747.s003.pdf]

**S1 File. Additional Materials and Methods.**

***Respiration of permeabilized cells.*** PC12 cells stably transfected with HTT exon1-$Q_{97}$ were plated in a XF24 cell culture plate and checked under the microscope for even seeding in complete growth media (Materials and Methods Cell Culture section). The day of the experiment the XF sensor cartridge was calibrated and loaded into the XF24 (Seahorse Bioscience) while cells were rinsed once with Mitochondria Assay Solution (MAS: Sucrose 70 mM, Mannitol 220 mM, $KH_2PO_4$ 5 mM, $MgCl_2$ 5 mM, HEPES 2 mM, EGTA 1mM, FA-free BSA 0.2% w/v). Cells were permeabilized with 500ul/well of MAS + digitonin (30 μM) for 10 minutes. The digitonin solution was then removed and 400 μL of MAS was added to all the wells before starting the experiment. Compounds in the four Seahorse XF ports contained: Port A: Succinate 5 mM plus ADP 0.25 mM, Port B: Olygomycin 2 μM, Port C: FCCP 4 μM, Antimycin A: 4 μM). See S1 Figure.

***Estimating Cellular HTT exon1 Levels by GFP Western Blot***. Levels of insoluble (sedimentable amyloid and inclusions) forms of HTT exon1 were determined using a GFP epitope in SDS-PAGE Western blots. Frozen PC12 cell pellets were resuspended and lysed with RIPA buffer (Sigma, R0278-50ML) plus protease inhibitors on ice, then centrifuged at 2,000 g and the pellets washed twice (resuspension followed by re-centrifugation) with lysis buffer. After removal the lysis buffer supernatant, pellets were dissolved in 100 μl formic acid, with overnight incubation at RT. Formic acid was removed by lyophilization and the resulting residue resuspended in 50 μL 10X PBS and 50 μL Laemmli Sample Buffer (BioRad, cat# 161-0737) with β-mercaptoethanol as reducing agent and boiling for 5 mins. Lysate aliquots containing 20 μg of total cell protein (representing ~340,000 PC12 cells) were loaded into wells of a TGX 4-15% gradient SDS-PAGE pre-cast gel (BioRad). After developing the SDS gels, their protein contents were electrotransferred onto a PVDF membrane for WB analysis. Within each Western blot, a GFP standard curve (Prospec, #pro-687-b) was also included (0.1, 0.05, 0.025, 0.01, and 0.005 μg of GFP) (example, S1 Figure, panel C). Membranes were probed with GFP antibody (CellSignaling, #2956S) 1:1000 and compatible IR-680 goat-anti-rabbit (Invitrogen) antibody was used for detection. Standard curves (S1 Figure, panel D) were constructed and the soluble and insoluble fractions of HTT calculated. Where blots of cell material showed less intense, faster moving, presumed fragments of HTT exon1-EGFP, their densities were included in the total to estimate cell levels of HTT exon1. Total protein assays were also conducted on aliquots of the gel loads to determine, as described above, the number of cells represented in each gel sample.
